# cIAPs Block Ripoptosome Formation, a RIP1/Caspase-8 Containing Intracellular Cell Death Complex Differentially Regulated by cFLIP Isoforms

**DOI:** 10.1016/j.molcel.2011.06.011

**Published:** 2011-08-05

**Authors:** Maria Feoktistova, Peter Geserick, Beate Kellert, Diana Panayotova Dimitrova, Claudia Langlais, Mike Hupe, Kelvin Cain, Marion MacFarlane, Georg Häcker, Martin Leverkus

**Affiliations:** 1 Section of Molecular Dermatology, Department of Dermatology, Venereology, and Allergology, Medical Faculty Mannheim, University Heidelberg, Heidelberg 68167, Germany; 2 Laboratory for Experimental Dermatology, Department of Dermatology and Venereology, Otto-von-Guericke-University Magdeburg, Magdeburg 39120, Germany; 3 MRC Toxicology Unit, Hodgkin Building, University of Leicester, P.O. Box 138, Lancaster Road, Leicester LE1 9HN, UK; 4 Department of Microbiology and Hygiene, University of Freiburg, Freiburg 79108, Germany

## Abstract

The intracellular regulation of cell death pathways by cIAPs has been enigmatic. Here we show that loss of cIAPs promotes the spontaneous formation of an intracellular platform that activates either apoptosis or necroptosis. This 2 MDa intracellular complex that we designate “Ripoptosome” is necessary but not sufficient for cell death. It contains RIP1, FADD, caspase-8, caspase-10, and caspase inhibitor cFLIP isoforms. cFLIP_L_ prevents Ripoptosome formation, whereas, intriguingly, cFLIP_S_ promotes Ripoptosome assembly. When cIAPs are absent, caspase activity is the “rheostat” that is controlled by cFLIP isoforms in the Ripoptosome and decides if cell death occurs by RIP3-dependent necroptosis or caspase-dependent apoptosis. RIP1 is the core component of the complex. As exemplified by our studies for TLR3 activation, our data argue that the Ripoptosome critically influences the outcome of membrane-bound receptor triggering. The differential quality of cell death mediated by the Ripoptosome may cause important pathophysiological consequences during inflammatory responses.

## Graphical abstract

**Graphical Abstract:**

**Figure:**
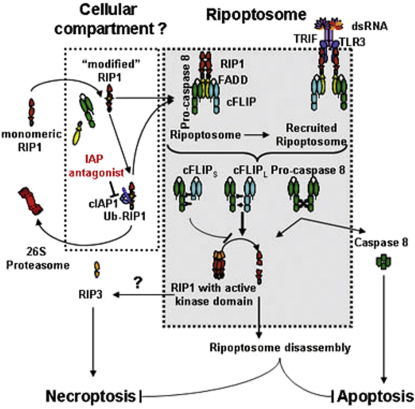

**Highlights:**

► Formation of the Ripoptosome, an intracellular death platform, is inhibited by cIAPs ► This high MW platform can be recruited to TLR3, leading to apoptosis or necroptosis ► cFLIP_S_ blocks caspase-8 activity in the complex required for disassembly/stability ► cFLIP_S_ promotes necroptosis in the absence of cIAPs

## Introduction

Cell death pathways are regulated at several different levels. Initiation points of these signals are organelles such as the mitochondria (Kelly and Strasser, 2011). Alternatively there are numerous examples of signaling platforms in cells named death-inducing signaling complex (DISC) (Lavrik et al., 2005), TNF complex II (Micheau and Tschopp, 2003), Apoptosome (Cain et al., 1999), PIDDosome (Tinel and Tschopp, 2004), toll-like receptor (TLR) complexes (Vercammen et al., 2008), or the RIG-I complex (Friedman et al., 2008; Rajput et al., 2011) that can initiate cell death. The longstanding observations that identical death stimuli such as TNF, TLR3 ligands, or death ligands may also activate programmed necrosis (necroptosis) depending on the cellular context complicate the understanding of cell death platforms (Holler et al., 2000; Matsumura et al., 2000; Vercammen et al., 1998). A contribution of another intracellular death platform containing RIP1 was proposed to be induced by excessive DNA damage requiring autocrine TNF secretion (Biton and Ashkenazi, 2011). Other studies showed that necroptosis induced by TNF requires receptor-interacting protein 3 (RIP3), which is phosphorylated and activated by RIP1 (Cho et al., 2009; He et al., 2009; Zhang et al., 2009). However, the regulation of apoptosis or necroptosis induced by a receptor-mediated or a genotoxic signal is far from being fully understood and is potentially of high clinical importance (Vandenabeele et al., 2010).

The largely endosomal TLR3 recognizes virus-derived double-stranded RNA or its synthetic homolog poly(I:C) and signals for inflammatory responses through NF-κB and type I interferon induction (Vercammen et al., 2008). As other membrane-bound receptors, TLR3 has the capacity to induce apoptosis in a TIR domain-containing adaptor-inducing interferon-β (TRIF)-dependent manner (Weber et al., 2010). The recruitment and binding of TRIF via the RIP homotypic interaction motif (RHIM) is required for the transduction of apoptotic signals (Kaiser and Offermann, 2005; Weber et al., 2010). However, the biochemical and molecular basis of TLR3-induced apoptosis or the propensity of this receptor to induce necroptosis has not been resolved to date. RIP1 also contains a C-terminal death domain (DD) and thereby interacts with the adaptor molecule Fas-associated death domain protein (FADD). In turn, FADD promotes recruitment of the initiator caspase-8 (for review, see Vercammen et al., 2008) by homotypic interaction of the death effector domains (DEDs) of both proteins. This signaling platform initiates cell death induction, tightly regulated by antiapoptotic proteins such as cellular FLICE-inhibitory protein (cFLIP). From the 11 known cFLIP isoforms, the long (cFLIP_L_) and the short (cFLIP_S_) isoforms are prominently expressed in cultured tumor cells (Budd et al., 2006).

A number of compounds that were originally designed to mimic binding of the mitochondrial protein Smac/DIABLO to the N-terminal IAP-binding motif (IBM) of XIAP (smac mimetics) also target cIAPs. These compounds induce rapid autoubiquitination and proteasomal degradation of cIAPs and are therefore rather IAP antagonists (Bertrand et al., 2008; Gaither et al., 2007; Petersen et al., 2007; Varfolomeev et al., 2007; Vince et al., 2007). More recently it was reported that cIAPs promote K11-ubiquitination of RIP1 and subsequent degradation, thereby protecting against TNF-induced cell death (Bertrand et al., 2008; Dynek et al., 2010). The molecular interplay of apoptotic and necroptotic signals has gained great attention by the very recent genetic evidence of RIP3-caspase-8 and RIP1-FADD double knockout mice. In these reports, loss of RIP3 or RIP1 protects from embryonic lethality caused by caspase-8 or FADD deficiency, respectively. These reports demonstrate the high relevance of these signaling modules for development (Kaiser et al., 2011; Oberst et al., 2011; Zhang et al., 2011) or, more recently, for T cell death (Ch'en et al., 2011) in mice. Nonetheless, the intracellular signaling platform that regulates apoptotic and necroptotic signals remains to be identified.

In this report we have characterized the intracellular signaling platform containing caspase-8, caspase-10, RIP1, FADD, and cFLIP isoforms. We demonstrate that loss of cIAPs promotes TLR3-induced RIP1-RIP3-mediated necroptosis. This signal is initiated at a 2 MDa complex distinct from TNF-induced complex II. To be consistent with other high Mw complexes, we termed this signaling platform “Ripoptosome,” based on the core scaffold function of RIP1. We then show that cells with low RIP1 or high cFLIP_L_ levels are resistant to Ripoptosome formation and therefore cell death. Intriguingly, in the absence of cIAPs, cFLIP_S_ was able to protect from Ripoptosome-induced apoptosis but promoted necroptosis that is caused by the lack of caspase activity within the complex. Cancer cells have the susceptibility to spontaneously form the Ripoptosome in response to IAP antagonists that may promote killing in a RIP1 kinase-dependent manner by necroptosis. Our study thus points to interesting therapeutic options for combining TLR agonists with IAP antagonists, with cFLIP isoforms representing critical regulators of the qualitative and quantitative responses from this complex.

## Results

### TLR3-Induced Cell Death Is Blocked by cIAPs and Proceeds in a Caspase- and RIP1-Kinase-Dependent Manner Independent from TNF Signaling

**Figure 1:**
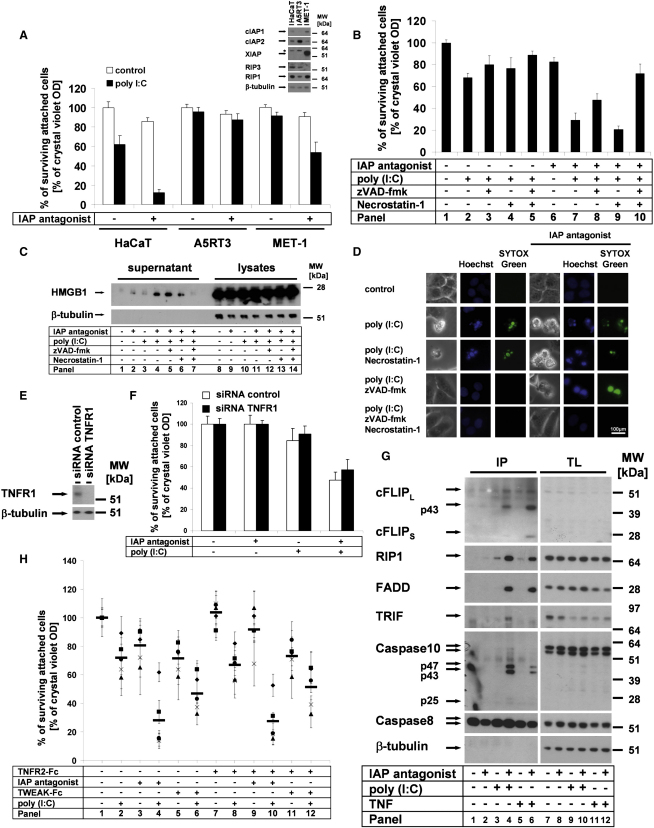
IAPs Protect Cells from TLR3-Induced Apoptosis and Necroptosis (A, B, F, and H) Cell viability was analyzed by crystal violet assay; average values of three independent experiments (±SEM) are shown. (A) HaCaT, A5RT3, and MET-1 cells were pretreated with IAP antagonist and subsequently stimulated with 50 μg/ml of poly(I:C). Inset shows analysis of cellular lysates by western blotting of the respective proteins. (B–D and H) Cells were stimulated as described in the Experimental Procedures. (B) In the absence of cIAPs, TLR3 ligation induces cell death in a caspase- and RIP1 kinase-dependent manner. (C) cIAPs suppress HMGB1 release upon TLR3 stimulation. HMGB1 release from HaCaT was detected by western blot analysis of cell-free supernatants and total cellular lysates. (D) cIAPs protect cells from TLR3 ligation-induced apoptosis and necroptosis. Cell death morphology of HaCaT cells was investigated by simultaneous staining with Hoechst-33342 and SYTOX Green, immediately followed by transmission and fluorescence microscopy, respectively (Supplemental Experimental Procedures). (E) TNFR1 knockdown was analyzed by western blot analysis in HaCaT cells transfected with control or TNFR1 siRNA. (F) TNFR1 knockdown does not alter TLR3-induced cell death. (G) Comparison of TLR3-induced caspase-8-containing complexes with TNF complex II. For analysis of caspase-8-containing complexes, caspase-8 coimmunoprecipitation (IP) was performed. Cells were prestimulated with IAP antagonist and subsequently stimulated with either poly(I:C) or TNF (10 μg/ml). (H) Primary human keratinocytes (from five different donors [different symbols; the horizontal bar represents the mean of all five donors examined]) are sensitized to TLR3-induced cell death in the absence of IAP function. Either IAP antagonist (panels 3–4) or TWEAK (panels 5–6) sensitizes to TLR3-induced cell death in a TNF-independent manner (panels 7–12).

We first tested whether cIAPs control TLR3-mediated cell death. IAP antagonist (compound A) (Vince et al., 2007) or TNF-like weak inducer of apoptosis (TWEAK) (Vince et al., 2008) induces rapid degradation of cIAP1 or cIAP2. Sensitivity to the artificial TLR3 ligand poly(I:C) upon treatment with IAP antagonist or TWEAK was studied in HaCaT keratinocytes, A5RT3, and MET-1 squamous cell carcinoma (SCC) cells. IAP antagonist promoted poly(I:C)-induced cell death of HaCaT and MET-1, but not of A5RT3 (Figure 1A; see Figures S1A and S1B available online). TWEAK treatment comparably sensitized HaCaT to TLR3 stimulation, whereas A5RT3 or MET-1 cells were not sensitized (Figures S1B and S1C). Knockdown of cIAP1, but not cIAP2, sensitized HaCaT cells to TLR3-induced cell death, revealing the decisive role of cIAP1. IAP antagonist further sensitized cIAP1 knockdown cells, indicating the contribution of cIAP2 to cell death resistance (Figure S1D). Next we tested the caspase inhibitor zVAD-fmk (zVAD) and the inhibitor of the kinase RIP1, Necrostatin-1 (Nec-1) (Degterev et al., 2008). Neither zVAD nor Nec-1 blocked poly(I:C)-induced cell death in the presence (Figure 1B, panels 2–4; Figure S1E, panels 2–4) or absence (Figure 1B, panels 7–9; Figure S1E, panels 7–9) of cIAPs. The combination of zVAD and Nec-1 largely rescued the cells (Figure 1B, panels 5 and 10; Figure S1E, panels 5 and 10). This suggests that simultaneous rescue from apoptosis and necroptosis is achieved only by the combined use of zVAD and Nec-1. Of note, although zVAD alone induces necroptosis in some cells such as L929 (for review, see Vandenabeele et al., 2010), in our cellular models of human epithelial cells this effect is not detected (compare Figure 4A; data not shown).

The release of high mobility group box 1 protein (HMGB-1) in the cellular supernatant is a characteristic of caspase-independent cell death (Scaffidi et al., 2002). In line with this finding, TLR3-induced release of HMGB-1 in the absence of cIAPs was unaffected by zVAD and marginally decreased by Nec-1 but completely suppressed by the combination of zVAD and Nec-1 (Figure 1C). We next analyzed cell death morphology by fluorescence microscopy studies. Typical apoptotic cells (membrane blebbing, DNA condensation) were detectable after poly(I:C) stimulation in the presence and absence of IAP antagonist (Figure 1D). Nec-1 did not alter the cell death phenotype. In the absence of cIAPs, zVAD modified cell death morphology (Figure 1D, right panels): we observed a more rounded shape and a lack of DNA condensation. Combined addition of zVAD and Nec-1, however, fully rescued the cells (Figure 1D, lowest panel). We conclude that in the absence of cIAPs and under conditions of caspase blockade, TLR3 stimulation results in RIP1-dependent necroptosis. IAP antagonist may induce autocrine production of TNF (Vince et al., 2007). Knockdown of TNFR1 (Figure 1E) or treatment with a number of soluble receptors (TNFR2-Fc, TRAILR2-Fc, and CD95-Fc) or the combination thereof did not significantly alter TLR3-mediated cell death (Figure 1F; Figure S1F). Taken together, our data demonstrate that cIAPs control TLR3-mediated apoptotic and necroptotic cell death independent from death ligand-induced autocrine loops. Importantly, our data using several different models of transformed epithelial cells demonstrate that such stress responses are highly dependent on the cellular context (e.g., regulated in a cell type-specific manner).

To tackle the molecular mechanisms responsible for the death signal, we performed immunoprecipitation (IP) of the caspase-8-interacting complex under various conditions (Figure 1G). cIAP depletion increased the interaction of caspase-8 with RIP1 upon TLR3 stimulation (Figure 1G, panel 4). TLR3-induced caspase-8 complex not only contained RIP1, FADD, the cFLIP cleavage fragment p43, and caspase-10 p47/p43 but also the TLR3 adaptor TRIF. In contrast, TRIF was absent in TNF-induced complex II (Figure 1G, panel 6), indicative of the specific recruitment of the complex to TLR3. To investigate the physiological relevance of our data in cell lines, we investigated the TLR3-induced cell death in primary human keratinocytes (PKs) (Figure 1H). These experiments demonstrated that loss of cIAPs combined with inactivation of XIAP promotes TLR3-induced cell death (Figure 1H, panels 4 and 6) independent from TNF signaling (Figure 1H, panels 7–12).

### cIAPs Block Spontaneous Ripoptosome Formation, a Complex that Modulates the Qualitative and Quantitative Cell Death Responses

**Figure 2:**
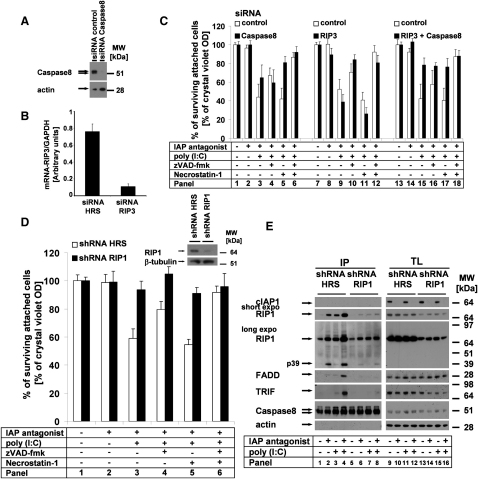
Increased Cell Death by Loss of cIAPs Is Regulated at the Ripoptosome; RIP1 Is Critical, whereas RIP3 or Caspase-8 Modulates the Quality of Cell Death (A) Caspase-8 siRNA is functional as determined by western blot analysis of caspase-8 protein. (B) The efficiency of RIP3 knockdown in HaCaT was analyzed by qPCR. The mean of two independent experiments (±SEM) is shown. (C and D) Cells were stimulated as described in the Experimental Procedures. Cell viability was analyzed by crystal violet assay; the mean of three independent experiments (±SEM) is shown. (C) The quality of cell death induced by TLR3 in the absence of cIAPs depends on caspase-8 or/and RIP3 expression. (D) Knockdown of RIP1 protects HaCaT from TLR3-induced cell death in the absence of cIAPs. Knockdown efficiency for RIP1 was analyzed by western blot analysis (inset). (E) RIP1 knockdown suppresses Ripoptosome formation. Caspase-8 immunoprecipitation (IP) was performed as outlined in the Experimental Procedures. Prolonged exposure of the blots reveals either caspase-8/RIP1 association in the unstimulated conditions or alternatively represents the background of the biochemical experiment.

We had observed two different phenotypes of TLR3-induced cell death and found molecules involved in either apoptosis or necroptosis in the same intracellular complex. We thus reasoned that both cell death responses are elicited at the same signaling platform. The next goal was to define the contribution of key proteins in IAP-controlled cell death pathway. Cell death was fully dependent on TLR3 expression and largely on TRIF (Figures S2A and S2B). Knockdown of caspase-8 (Figure 2A) resulted in partial suppression of TLR3-induced cell death in the absence of cIAPs that was largely rescued by Nec-1, but not by zVAD (Figure 2C, panels 3–5). This finding indicated that caspase-8 is contributing to the execution of the apoptotic cell death in the absence of cIAPs, as previously suggested (Wang et al., 2008). Knockdown of RIP3 (Figure 2B) did not result in protection from TLR3-induced cell death. However, cell death was largely blocked by zVAD independent from Nec-1 treatment (Figure 2C, panels 9–12), indicating that RIP3 determines necroptotic cell death in our cellular systems. Thus, loss of RIP3 disables TLR3-mediated necroptosis without affecting the apoptotic response. Combined knockdown of RIP3 and caspase-8 largely protected cells from cell death altogether (Figure 2C, panels 15–18), indicating that caspase-8 and RIP3 are the two critical effectors of TLR3-induced cell death in the absence of IAPs. We next investigated the function of RIP1. Knockdown of RIP1 (Figure 2D, inset) resulted in full protection from TLR3-IAP antagonist (Figure 2D and Figure S2C) or TLR3-TWEAK-mediated cell death (Figure S2D). Having defined the decisive role of RIP1 for TLR3-mediated cell death in the absence of cIAPs, we next characterized the caspase-8-interacting signaling platform by IP. These experiments revealed that treatment with IAP antagonist alone resulted in caspase-8-RIP1-FADD interaction (Figure 2E, panel 2) which is insufficient for cell death induction (compare Figure 2D, panel 2). Additional stimulation with poly(I:C) resulted in formation of a complex that also included TRIF (Figure 2E, panels 3–4). Knockdown of RIP1 interfered with complex formation under all conditions (Figure 2E, panels 5–8). These data demonstrate that RIP1 is decisive for the activation and formation of the caspase-8-RIP1 signaling complex which we thus designate “Ripoptosome.”

### cFLIP Is a Critical Regulator of the Ripoptosome

**Figure 3:**
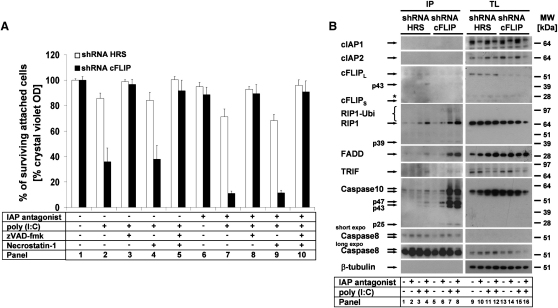
cFLIP Suppresses Ripoptosome Formation and Apoptosis in the Absence of cIAPs (A) cFLIP protects from TLR3-induced cell death. A5RT3 cells were transduced with control shRNA (HRS) or cFLIP shRNA and stimulated as described in the Experimental Procedures. Cell viability was analyzed by crystal violet assay; the mean of three independent experiments (±SEM) is shown. (B) cFLIP suppresses Ripoptosome formation in A5RT3 cells. Ripoptosome formation was studied by caspase-8 IP. Note the dramatic accumulation of caspase-10 upon cFLIP knockdown.

We had noted that A5RT3 cells were not sensitized to TLR3-induced cell death by IAP antagonist or TWEAK (Figure 1A). Since cFLIP regulates caspase-8-dependent cell death, we next studied the impact of this molecule for TLR3-mediated cell death. Retroviral infection with a control construct resulted in a minor sensitization to TLR3 ligation (Figure 3A, white bars). However, knockdown of cFLIP resulted in a major sensitization to TLR3-induced death (Figure 3A, panel 2), further increased by depletion of cIAPs (Figure 3A, panel 7). Inhibitor studies in these cells revealed that cell death was largely apoptotic (Figure 3A, panel 3 or 8). When we studied Ripoptosome formation in these cells, A5RT3 lacking cFLIP showed spontaneous Ripoptosome formation in the absence of cIAPs (Figure 3B, panel 6) with further increase upon TLR3 stimulation (Figure 3B, panel 7–8). Interestingly, downregulation of cFLIP led to accumulation of large amounts of caspase-10 in the Ripoptosome. These data argue that cFLIP controls Ripoptosome formation by blocking the association of FADD, RIP1, and caspase-10 with caspase-8.

### cFLIP Isoforms Differentially Regulate Poly(I:C)-Induced Cell Death by Regulation of the Ripoptosome

**Figure 4:**
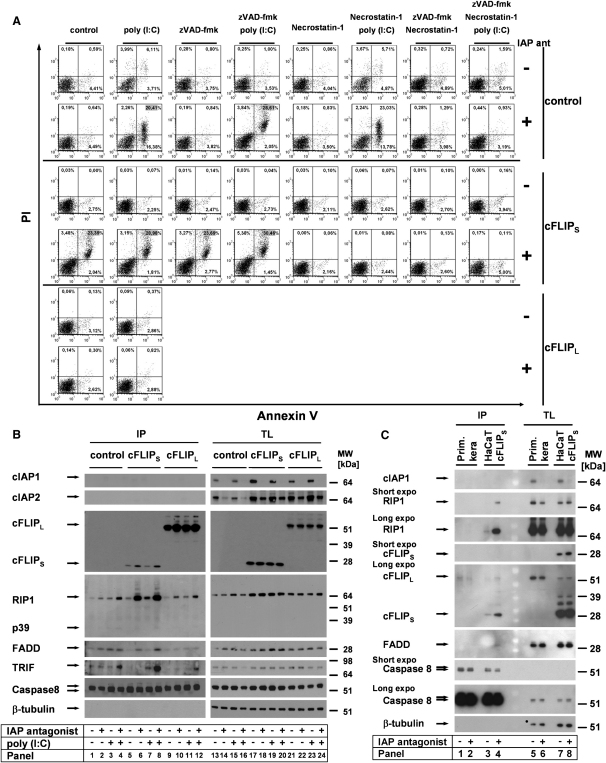
cFLIP Isoforms Differentially Regulate Ripoptosome Formation and Modulate the Quality of Cell Death in the Absence of cIAPs (A) The quality of cell death is differentially regulated by cFLIP isoforms. HaCaT cells were transduced with cFLIP_L_, cFLIP_S_, or vector alone and stimulated as indicated in the Experimental Procedures. Cell death was characterized by Annexin V/PI double staining, followed by FACS analysis. (B) Ripoptosome formation is modulated by cFLIP isoforms in a differential manner. Ripoptosome was precipitated by caspase-8 Abs. (C) Absence of spontaneous Ripoptosome formation in PK upon IAP antagonist treatment when compared to HaCaT-cFLIPs.

Next we investigated functional differences of cFLIP isoforms expressed in human cells. We thus overexpressed cFLIP_L_ or cFLIP_S_ in HaCaT which showed low endogenous cFLIP expression (Diessenbacher et al., 2008). We then studied quantitative and qualitative cell death responses to TLR3 stimulation by Annexin V-PI staining (Figure 4A) and crystal violet assays (Figures S3A and S3B). Loss of cIAPs promoted TLR3-induced cell death in vector controls (Figure 4A) independent from RIP1-kinase activity (Figure 4A, Figures S3A and S3B, panel 5). zVAD protected cells from apoptotic cell death (concluded from the absence of Annexin V+/PI− cells) but only marginally protected from cell death (Figure 4A, control cells). These data closely correlated with our crystal violet results (Figures S3A and S3B, white bars). Combination of Nec-1 and zVAD entirely blocked cell death. cFLIP_L_ completely protected from poly(I:C)-induced cell death under all conditions (Figure 4A, Figures S3A and S3B, black bars). In contrast, loss of cIAPs resulted in spontaneous cell death in HaCaT-cFLIP_S_, and the outcome of additional TLR3 ligation was minor (Figure 4A, Figures S3A and S3B, gray bars). Remarkably, this cell death was solely RIP1 kinase dependent, whereas zVAD had no protective effect (Figure 4A, Figures S3A and S3B, compare panels 4 and 5, gray bars). These data show that cFLIP_S_ profoundly changes the qualitative response to TLR3 ligation whenever cIAPs are absent. We next studied Ripoptosome formation in these cells. Loss of cIAPs in HaCaT-cFLIP_S_ increased RIP1 association with the complex (Figure 4B, panels 4 and 8). Interestingly, treatment with IAP antagonist alone was sufficient to induce spontaneous complex formation in HaCaT-cFLIP_S_, although TRIF was not associated (Figure 4B, panel 6). cFLIP_L_ suppressed the increased association of RIP1 or FADD with the complex upon IAP antagonist/TLR3 stimulation (Figure 4B, panels 10 and 12). Of interest, total levels of RIP1 slightly increased in HaCaT-cFLIP_S_. To investigate if Ripoptosome formation is a more general phenomenon, we next studied HeLa cells. HeLa cells were similarly sensitized to TLR3 cell death by loss of IAP function (Figure S3C, panel 3). Inhibitor studies revealed that cell death proceeds in a purely apoptotic manner (Figure S3C, panel 4), most likely due to the lack of RIP3 expression (Figure S3D). In line with this assumption, ectopic expression of wild-type RIP3 (RIP3 WT) but not a kinase deficient mutant of RIP3 (RIP3 KD; Cho et al., 2009) in HeLa cells led to increased caspase-independent TLR3-induced cell death in the absence of IAPs (Figure S3F). In contrast to the data in HaCaT, HeLa cells were largely protected from TLR3-induced cell death by both cFLIP isoforms. cFLIP_S_-expressing cells were not sensitive to IAP antagonist alone (Figure S3C, black and gray bars). When we studied Ripoptosome formation in these cells, cFLIP_L_ but not cFLIP_S_ was inhibitory, similar to HaCaT (Figure S3E, compare panels 2, 6, and 10). This effect was particularly evident because RIP1 was strongly induced by overexpression of both cFLIP isoforms in HeLa (Figures S3E, TL). To investigate the formation of the Ripoptosome under more physiological conditions, we studied its formation in PKs (Figure 4C). Whereas HaCaT-cFLIPs spontaneously formed the Ripoptosome it was not detectable in PK in the absence of IAP function (Figure 4C). This finding might be explained by high levels of cFLIP_L_ expression in PK (Kavuri et al., 2011; Leverkus et al., 2000) (Figure 4C, panels 5 and 6 versus 7–8) and argues that spontaneous Ripoptosome formation is highly cell type specific and mainly occurring in transformed cells in a cFLIP isoform-dependent manner. Taken together, our data suggest that cFLIP isoforms regulate Ripoptosome formation in opposite directions and that the caspase-like domain of cFLIP_L_ inhibits Ripoptosome formation or its stability. It remains to be determined how the stoichiometric ratio between cFLIP_L_ and cFLIP_S_ impacts the physiological or pathophysiological signaling. Our studies using large differences in stoichiometry of the different cFLIP isoforms in this context serve as “proof-of-principle” studies.

### Promotion of Necroptosis by cFLIP_S_ in the Absence of IAPs Is Independent of TNFR1 Signaling and Occurs in a 2 MDa Complex

**Figure 5:**
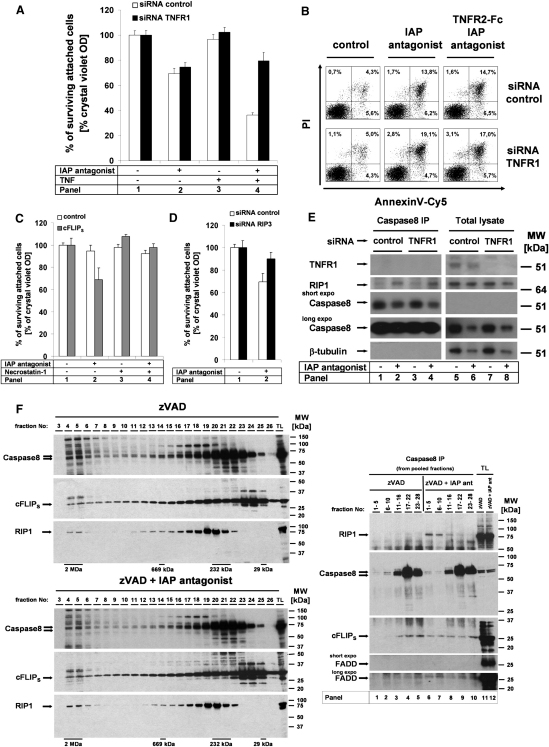
cFLIP_S_ Promotes Spontaneous Ripoptosome Formation, a 2 MDa Complex, in the Absence of cIAPs; Ripoptosome-Induced Necroptosis Proceeds in a RIP1 Kinase- and RIP3-Dependent Manner Independent from TNF Signaling (A, B, and E) cFLIP_S_-HaCaT were transfected with control or TNFR1 siRNA. (A, C, and D) Cell viability was analyzed by crystal violet assay; the mean of three independent experiments (±SEM) is shown. (A) Spontaneous cell death induced by cIAPs depletion in cFLIP_S_ cells is independent from TNFR1. siRNA-transfected cells were prestimulated with IAP antagonist followed by stimulation with TNF (100 ng/ml). (B) Spontaneous cell death of HaCaT-cFLIP_S_ in the presence of IAP antagonist is independent from autocrine TNF signaling. Cells were stimulated with IAP antagonist and TNFR2-Fc, respectively. Cell death was determined by Annexin V/PI double staining followed by FACS analysis. (C) Spontaneous cell death induced by cIAPs depletion in HaCaT-cFLIP_S_ is RIP1 kinase dependent. (D) Spontaneous cell death induced by cIAP depletion in HaCaT-cFLIP_S_ is RIP3 dependent. HaCaT-cFLIP_S_ were transfected with RIP3 siRNA or control siRNA and then treated with IAP antagonist. (E) Spontaneous Ripoptosome formation in HaCaT-cFLIP_S_ in the absence of cIAPs is independent from TNFR1 signaling. Ripoptosome formation was analyzed by caspase-8 IP in the presence of zVAD from 2 × 10^6^ transfected cells 4 hr after IAP antagonist stimulation (200 nM). (F) Inhibition of cIAPs promotes Ripoptosome formation, a 2 MDa complex. HaCaT-cFLIP_S_ were either prestimulated with zVAD (10 μM) alone or in combination with IAP antagonist (200 nM) for 4 hr. Cellular lysates were used for chromatographic gel filtration analysis. Numbers indicate fractions collected. Fractions 3–26 were analyzed by western blotting (left panels) for the respective proteins. All fractions were subsequently pooled as indicated, and caspase-8 IP was performed as described in the Experimental Procedures. Proteins found in different pooled fractions were analyzed by western blotting.

To dissect the molecular mechanism of the differential effect of cFLIP isoforms, we next investigated spontaneous cell death occurring in HaCaT-cFLIP_S_ upon cIAP depletion. We first studied if autocrine TNF signaling might explain this phenomenon. Therefore we knocked down TNFR1, which did not alter cell death induced by IAP antagonist alone (Figure 5A, panel 2). Of note, increased cell death by exogenous addition of TNF was fully blocked by TNFR1 knockdown, whereas sensitization by addition of IAP antagonist was unaltered (Figure 5A, panel 4). Annexin V/PI staining revealed that cells died within 8 hr without appearance of a predominant Annexin V+/PI− cell population (Figure S4A). Ripoptosome formation preceded the first detection of cell death and persisted until 12–18 hr after inactivation of IAPs (Figure S4B). TNFR1 knockdown (Figure 5B, right panel) and/or blockade of autocrine TNF (Figure S4A, right panel) also did not protect from cell death. In contrast, Nec-1 (Figure 5C, panel 4) or RIP3 knockdown (Figure 5D) fully blocked IAP antagonist-induced cell death in HaCaT-cFLIP_S_. To exclude the possibility that Ripoptosome formation is mediated by TNF, we performed caspase-8 IP in TNFR1 knockdown cells. Despite efficient knockdown of TNFR1 (Figure 5E, total lysate), caspase-8-RIP1 interaction was unchanged (Figure 5E, panels 2 and 4). Moreover, TNF complex I and II analysis in HaCaT in the absence of IAPs excluded the possibility that TNFR1 recruits caspase-8 via RIP-1 to the receptor (Figure S4C, panels 3–4). These data argue that recruitment of RIP1 to the TNFR1 complex I is independent from Ripoptosome formation and most likely represents a different pool of RIP1 than present in the Ripoptosome. Consequently it cannot be called TNF complex II.

To investigate the composition of the native RIP1-caspase-8 interaction platform in more detail, we perfomed gel filtration analysis. RIP1 and caspase-8 were mainly detected in their monomeric form (Figure 5F, left panels; RIP1, fractions 18–21; caspase-8, fractions 20–23), indicating that only a small amount of total cellular RIP1 and caspase-8 interacts. This is clearly evident since both proteins were detected in fractions corresponding to a complex of 2 MDa (Figure 5F, left panels, fractions 4–6). We next performed caspase-8 IP, using pooled samples from the column fractions. Interestingly, these experiments showed that upon IAP degradation caspase-8/RIP1/cFLIP interaction occurred in the 2 MDa complex but not in the lower MW fractions (Figure 5F, right panel; compare panels 6–10 with panels 1–5). Our data argue that cIAPs block spontaneous Ripoptosome formation in the 2 MDa complex and that other thus far unknown molecules are present in this 2 MDa complex. Taken together, our data argue that the Ripoptosome is a large intracellular complex that is negatively regulated by cIAPs and that its formation is necessary for cell death execution. cFLIP_S_ deviates cell death to RIP3-dependent necroptosis in a TNF-independent manner in the Ripoptosome.

### cFLIP_S_ but Not cFLIP_L_ Blocks Caspase-8 Activity within the Ripoptosome

**Figure 6:**
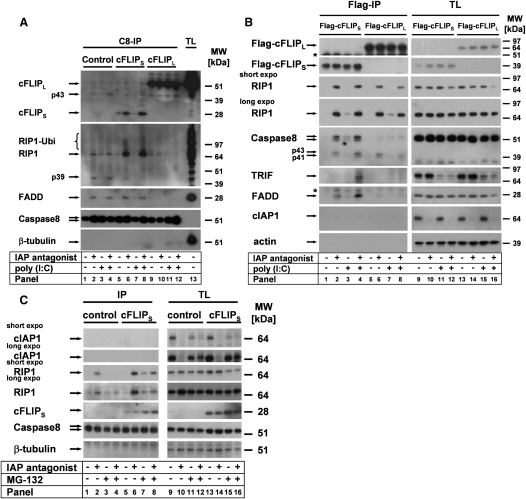
Caspase Activity in the Ripoptosome Is Differentially Regulated by cFLIP Isoforms and Is Negatively Regulated by Proteasome Function (A and C) Ripoptosome formation was studied by caspase-8 IP. (A) cFLIP isoforms differentially regulate caspase activity in the Ripoptosome. HaCaT-cFLIP_S_ cells were stimulated with IAP antagonist and zVAD for 1 hr with poly(I:C) or diluent alone. (B) In the absence of cIAPs, cFLIP_S_ but not cFLIP_L_ blocks caspase-8 activity in the Ripoptosome. HaCaT cells were transduced with retroviral vectors containing Flag-tagged cFLIP_L_ or cFLIP_S_. Cells were prestimulated with IAP antagonist alone or subsequently with poly(I:C), followed by anti-Flag IP. Asterisks indicate nonspecific binding of Abs. (C) Ripoptosome formation is negatively regulated by proteasome function in HaCaT-cFLIP_S_. Cells were prestimulated with MG132 for 10 min followed by stimulation with IAP antagonist for 4 hr. Experiments shown in (A)–(C) were performed in the presence of zVAD (10 μM).

To get further mechanistic insight into how the Ripoptosome activates apoptotic and necroptotic death, we decided to study enzymatic activity within the native complex utilizing complex-associated proteins as substrates. As shown in Figure 6A, HaCaT-cFLIP_S_ cells formed a strong Ripoptosome containing uncleaved RIP1 (Figure 6A, panels 6 and 8). In contrast, control cells contained comparable amounts of uncleaved and cleaved RIP1 (p39), indicative of caspase activity within the complex (Figure 6A, panels 2 and 4). In line with the suggestion of Cho et al. (Cho et al., 2009), our data indicate that caspase activity within the Ripoptosome blocks necroptosis by caspase-mediated RIP1 cleavage. Due to the fact that large amounts of caspase-8 are uncomplexed (Figure 5F), we wanted to extend our studies with a different bait for the IP of the complex. We thus established cell lines expressing Flag-tagged cFLIP isoforms. The response to IAP antagonist and TLR3 ligation was similar to cells expressing untagged cFLIP isoforms (data not shown). We precipitated the Ripoptosome using IP against the Flag-tag (Figure 6B). In the absence of cIAPs, cFLIP_S_ was complexed with RIP1, FADD, and, after TLR3 ligation, TRIF, whereas cFLIP_L_ exhibited less complex formation. Intriguingly, the Ripoptosome of cFLIP_L_-expressing cells mostly contained cleaved caspase-8, indicative of catalytic activity of caspase-8-cFLIP_L_ heterodimers (Oberst et al., 2010; Wachter et al., 2004). In contrast, the Ripoptosome of cFLIP_S_-expressing cells mainly contained uncleaved caspase-8, suggesting that the catalytic activity in the complex is repressed.

The question arose as to how cIAPs prevent spontaneous Ripoptosome formation. One likely explanation is degradative ubiquitination of RIP1 by cIAP1, as shown for K11-linked polyubiquitination (Dynek et al., 2010) or the constant K48-linked degradation in tumor cells (Bertrand et al., 2008). We thus studied complex formation in the presence of the proteasomal inhibitor MG-132. IAP antagonist-induced cIAP1 degradation was blocked by MG-132 (compare Figure 6C, lane 10 versus 12; lane 14 versus 16). Interestingly, MG-132 treatment alone facilitated formation of the Ripoptosome (Figure 6C, panel 5 versus 7). Moreover, degradation of cIAPs correlated with increased RIP1 protein levels (Figure S3E, panels 14–15) together with spontaneous Ripoptosome formation in HeLa cells (Figure S3E, panel 2). Lastly, the simultaneous treatment with IAP antagonist and MG-132 suppressed Ripoptosome formation (Figure 6C, compare panel 2 with 4 and panel 6 with 8). Of note, formation of the Ripoptosome in the presence of IAP antagonist and MG-132 occurs despite the fact that MG-132 decreases total RIP1 levels (Figure 6C, compare panels 9–10 with 11–12, and panels 13–14 with 15–16). Taken together, these data suggest that cIAPs constantly prevent Ripoptosome formation and stabilization by ubiquitination and subsequent proteasomal degradation of a fraction of RIP1, at least in tumor cell lines. Future studies are required to corroborate this hypothesis in more detail.

## Discussion

A number of different molecular platforms have been shown to contain the kinase RIP1. Some of these platforms (such as TNFR1 complex I; NOD complex) activate NF-κB, whereas others (TNF complex II; TRAIL or CD95 DISC) signal for cell death (Declercq et al., 2009). In this report we have described an intracellular complex that integrates different upstream stress signals converging at the kinase RIP1. Loss of cIAPs allows for caspase-dependent and RIP1-dependent cell death initiated at this complex. Consequently, it has the ability to induce both apoptosis and necroptosis based on the expression and activity of the different molecular components. These include RIP1, FADD, caspase-8, caspase-10, and cFLIP isoforms. Although we show that cFLIP negatively regulates the recruitment of caspase-10 or FADD into the complex, the biological significance of caspase-10 or its activity to the assembly or activity of the complex is to date unclear and is under current investigation. We have studied the complex in three different epithelial cell lines, arguing that formation of the complex is a common event. Since RIP1 proved to be indispensible for the formation of this complex, we suggest calling it “Ripoptosome.” Our data clearly show that the qualitative response to Ripoptosome activation is highly cell type specific and depends on the stoichiometry of the molecular components. This notion is supported by our RIP3 overexpression studies in HeLa cells. HeLa undergo necroptosis upon cIAP depletion whenever RIP3 is present in these cells. The data support the previous findings of the crucial relevance of RIP3 kinase function for the execution of necroptosis (Cho et al., 2009). Taken together, our data demonstrate the important contribution of the Ripoptosome to the qualitative and quantitative responses upon membrane-bound receptor triggering.

**Figure 7:**
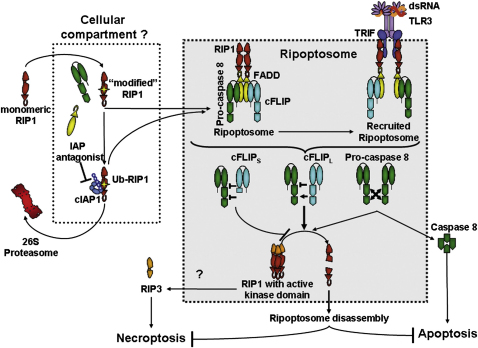
Working Model of Activation and Signaling by the Ripoptosome and Its Regulation by cFLIP Isoforms A fraction of RIP1 is constantly “modified” (likely by cellular stress signals) to an active conformation. cIAPs ubiquitinate “modified” RIP1 targeting it to degradation by the 26S proteasome. The unknown cellular compartment contains not only “modified” RIP1 but also caspase-8, FADD, and cFLIP. Addition of IAP antagonist leads to autoubiquitination of cIAPs and degradation. This is the signal for the formation of the Ripoptosome, a complex of “modified” RIP1, FADD, caspase-8/10, and cFLIP isoforms. The Ripoptosome can be recruited to RHIM-binding modules such as TRIF, thereby linking it to TLR3. Depending on the composition of the Ripoptosome, it activates apoptosis and/or necroptosis. Procaspase-8 homodimers within the Ripoptosome form active caspase-8 and subsequent apoptosis. cFLIP_L_-caspase-8 heterodimers within the Ripoptosome have enzymatic activity in the complex insufficient to generate active caspase-8 heterotetramers necessary for apoptosis induction, although sufficient for RIP1 cleavage. Caspase-8-cFLIP_S_ heterodimer lacks proteolytic activity that is required not only for full caspase-8 activation but also for RIP1 degradation. Thus RIP1 accumulates in the Ripoptosome and initiates necroptosis via RIP3 in a RIP1 kinase-dependent manner.

Spontaneous formation of the Ripoptosome is suppressed by the E3 ubiquitin ligases cIAP1 or -2 that induce RIP1 ubiquitination and degradation. This conclusion is based on our studies using proteasomal inhibitor MG-132. Our data furthermore suggest that the Ripoptosome is necessary but insufficient for induction of cell death and is disassembled by complex-intrinsic caspase activity, most likely by RIP1 cleavage (Figure 7). This is supported by previous data showing that the caspase inhibitor zVAD increases caspase-8-RIP1 complex stability or, potentially, formation (Geserick et al., 2009; Wang et al., 2008). When caspase activity in the complex is low, RIP1 kinase/RIP3-dependent necroptosis is the consequence. Importantly, we have excluded the possibility that the Ripoptosome is a mere secondary TNF complex II (Micheau and Tschopp, 2003) induced by autocrine TNF or other death ligands. This notion is supported by our data showing that spontaneous Ripoptosome formation was not altered upon TNFR1 knockdown.

Caspase-8-interacting platforms have been largely investigated in respect to death receptor signaling. Our studies of TLR3-induced cell death give biochemical evidence that a death signaling platform interacts in a stimulus-dependent manner with TRIF, the sole adaptor of TLR3. Recently, caspase-8-mediated cleavage of RIP1 was demonstrated to negatively regulate the activation of IRF3 in the RIG-I complex, arguing for a central role of RIP1 and caspase-8 not only for cell death but also for inflammatory or antiviral responses (Rajput et al., 2011). In the absence of cIAPs the Ripoptosome spontaneously forms and presumably facilitates subsequent Ripoptosome recruitment to TRIF. These data together with our previous study (Geserick et al., 2009) indicate that the Ripoptosome can be recruited to different receptors or their adaptors. We propose that this interaction may be achieved either via RHIM or the DD of RIP1. Of note, RIP1 is found in TNF complex I, although caspase-8 is activated downstream of TNF complex I (Harper et al., 2003; Micheau and Tschopp, 2003). Adding to this point, the absence of cIAPs does dramatically alter RIP1 modification in complex I, but this modification of RIP1 is not associated with recruitment of caspase-8 to TNF complex I (compare Figure S4C). We speculate that RIP1 recruited to TNFR1 does not necessarily represent the same pool of RIP1 found in the Ripoptosome and does not transit to complex II. Rather, TNF complex II might associate with the Ripoptosome whenever IAPs are absent. Future studies are required to delineate this point in more detail.

To further understand the role of caspase activation in Ripoptosome signaling that is limited when solely using pharmacological inhibitors, we studied the impact of cFLIP. Our data show that cFLIP isoforms differentially regulate formation and activity of the Ripoptosome. It is noteworthy that both cFLIP isoforms suppressed TLR3-triggered apoptotic, but not necroptotic cell death. cFLIP_L_ forms heterodimers with caspase-8 that have enzymatic activity but are unable to form fully active heterotetramers of caspase-8 (Boatright et al., 2004). The caspase-8/cFLIP_L_ heterodimers cleave only selected substrates (Pop et al., 2011). We suggest that one of the key substrates within the Ripoptosome is RIP1. Cleavage of RIP1 (the main scaffold of the Ripoptosome) may then promote disassembly of the complex, as previously suggested for TNF complex II (Cho et al., 2009). This needs to be formally proven in the future, although the very recent genetic evidence underlines the physiological relevance of our studies in vivo (Oberst et al., 2011). In contrast, cFLIP_S_-caspase-8 heterodimer is predicted to not have enzymatic activity. This notion is supported by our data (Figure 6A), in which we show that RIP1 cleavage within the Ripoptosome is achieved even in the presence of zVAD, whereas it is fully blocked by cFLIP_S_. In line with this finding, expression of cFLIP_S_ is associated with increased levels of RIP1 in the Ripoptosome, sufficient for RIP3 activation and thereby necroptosis. Of note, we were unable to detect RIP3 within the Ripoptosome, possibly due to the lack of specific reagents for biochemical analysis. Therefore, we currently cannot conclusively prove the point that RIP3 is recruited to Ripoptosome, although it was described in the TNF complex II of Jurkat T cells (Cho et al., 2009). Summing up these data, our report suggests that cFLIP isoforms are the critical “rheostat” of caspase activity in the Ripoptosome, regulating the quality of cell death. It remains to be answered how different stoichiometric quantities of cFLIP_L_ and cFLIP_S_ may influence the qualitative outcome of Ripoptosome-induced cell death. This intriguing question merits future experimental studies, likely by using in ex vivo systems developed for the study of death receptor-associated complexes (Hughes et al., 2009). Interestingly, inhibition of RIP1 kinase function with Nec-1 also promotes complex disassembly or blocks its assembly (Geserick et al., 2009). We speculate that RIP1 autophosphorylation might be required for Ripoptosome stabilization. Clearly, future studies about the role of different RIP1 phosphorylation sites will undoubtedly be of high interest.

One important issue is the mechanism whereby cIAPs impact on Ripoptosome formation. Our chromatographic gel filtration assay gave important insights. First, the amount of RIP1, caspase-8, and FADD associated with the Ripoptosome in HaCaT cells is rather small. Second, the uncomplexed components of the Ripoptosome studied thus far were found in the 2 MDa range before inactivation of cIAPs. We speculate that the part of RIP1 associated with the 2 MDa fractions defines the amount of RIP1 capable to form the Ripoptosome. The question of whether the translocation of RIP1 is another mechanism of assembly of the complex, as indicated by the RIP1 overexpression studies by Tenev et al. (Tenev et al., 2011, in this issue of *Molecular Cell*), needs to be clarified in future studies. In our cellular model, specific interaction of endogenous RIP1 and caspase-8 is seen only upon degradation of cIAPs in these fractions. It is currently unclear what differentiates RIP1 associated with the 2 MDa fractions from monomeric RIP1. We speculate that stress-associated posttranslational modification (e.g., phosphorylation), binding of additional (known or unknown) proteins, the transfer of RIP1 to a cellular compartment to be identified, or all of the above named reasons are necessary. We suggest that the fraction of RIP1 eluting with the 2 MDa fractions could represent this compartmentalized/modified RIP1. We further hypothesize that cIAPs only target the modified RIP1 for ubiquitination (likely at K377; O'Donnell et al., 2007) and proteasomal degradation. This explains why certain cell types (in which large fractions of RIP1 are accessible to cIAPs [e.g., HeLa, compare Figure S3E, panel 15]) show higher RIP1 protein levels upon treatment with IAP antagonist, whereas others (HaCaT) do not exhibit this effect. IAP antagonists cause rapid autoubiquitination and subsequent proteasomal degradation of cIAPs (Bertrand et al., 2008; Petersen et al., 2007; Varfolomeev et al., 2007; Vince et al., 2007). When we studied Ripoptosome formation in the presence of a proteasomal inhibitor, we found that the Ripoptosome spontaneously forms in the presence of IAPs whenever the 26S proteasome is blocked. This suggests that it is not the degradation of cIAPs that is a prerequisite for Ripoptosome formation. Rather, we suggest that it is cIAP-mediated degradation of compartmentalized RIP1 that precludes Ripoptosome signaling (Figure 7). Based upon the MG132 studies, another intriguing aspect was noted. cIAPs are degraded upon blockade of the proteasome. These results suggest that another degradative pathway is involved in the control of cIAP levels in the cell. We hypothesize that this degradative pathway involves the lysosome, as it was already shown that cIAPs can be targeted to lysosomes by TWEAK (Vince et al., 2008; Wicovsky et al., 2009). An elegant recent study has investigated the nature of ubiquitin chains attached to RIP1 in vivo using mass spectroscopy of complex-associated proteins (Gerlach et al., 2011). It was found that in contrast to the rather monomorphous linear ubiquitin modification of NEMO, the RIP1 ubiquitination is highly complex and contains numerous different types of ubiquitin chains in vivo (e.g., K48, K11, linear [M1], and K63), at least in TNF complexes (Gerlach et al., 2011). Undoubtedly it will be intriguing to study the nature of ubiquitin modifications of RIP1 and the functional impact of cIAPs more directly in the Ripoptosome.

The effector function of proapoptotic molecules such as caspase-8 or FADD is very well established. The mechanistic involvement of FADD, RIP1, caspase-8, or cFLIP in nonapoptotic signals has been a matter of intense debate over the past decade. In vivo evidence for the relevance of these nonapoptotic signals was limited, hampered by the embryonic lethality of caspase-8, cFLIP, or FADD knockout mice. Several very recent reports have provided compelling evidence that the absence of core Ripoptosome components (namely FADD and caspase-8) is lethal due to augmented RIP1-RIP3-dependent necroptosis (Kaiser et al., 2011; Oberst et al., 2011; Zhang et al., 2011). The molecular mechanism of the Ripoptosome that we propose in this report explains the phenotype observed in the genetic models. RIP1 knockout mice are viable due to the central role of this molecule in Ripoptosome formation. When FADD or caspase-8 are absent, the disassembly of the Ripoptosome is impossible, because FADD cannot recruit caspase-8 necessary for RIP1 degradation. The absence of RIP3 blocks the effector phase of necroptosis downstream (or within) the Ripoptosome. Certainly additional studies are needed that involve experiments about the differential role of cFLIP in mice, although this is challenging due to the fact that genes encoding cFLIP and caspase-8 are closely clustered.

Viruses, such as the Kaposi's sarcoma-associated herpes virus (KSHV), can confer apoptosis resistance and tumor progression by expression of vFLIP isoforms that resemble cFLIP_S_ (Punj et al., 2009) and are upregulated during late stages of KHSV-induced sarcoma (Sturzl et al., 1999). Our current findings that show that cells expressing high levels of cFLIP_S_ initiate necroptosis upon cIAP depletion may prove to be of high pathophysiological relevance. cIAPs may be critically required to control virus-induced infections, tumor development, or alternatively the host response influenced by either apoptotic or necrotic cell death (Challa et al., 2010). TLR3 is widely expressed on tumor cells (Salaun et al., 2007), and its signals affect a wide range of physiological and pathophysiological processes (Huang et al., 2008; Suhir and Etzioni, 2010). The negative regulation of the Ripoptosome by cIAPs as suggested in this report could also influence the immune response against tumors. Existing evidence suggests that TLR3 ligands can be used as anticancer agents and that a combination of CD40 and TLR agonists may synergistically act as immunostimulants by suppression of tumor growth in mice (Stone et al., 2009). Expression of cIAPs by tumor cells may thus be required to evade the tumor immune response. Antagonists of IAP function such as those used in this study may therefore represent a potential antitumor strategy to overcome necrosis resistance activated by TLR3 agonists for instance in Epstein-Barr virus (EBV)-induced epithelial cancers (nasopharyngeal carcinoma) (Iwakiri et al., 2009). In turn, this may lead to increased immune responses favoring tumor elimination. The intriguing pathophysiological role of cIAP/RIP1 regulation during TLR3 signaling leading to Ripoptosome activation, as demonstrated in this report, requires further intense investigation in the future. Since transformation of PK favors a necrotic response, while “contracting” the apoptotic response to chemotherapeutics (Kravchenko-Balasha et al., 2009), tight control of the Ripoptosome by IAP proteins could prevent successful treatment of epithelial malignancies such as cervical cancer. Understanding the assembly, regulation, and degradation of the Ripoptosome in more detail will likely open up avenues for tumor therapy.

## Experimental Procedures

### Cytotoxicity Assays

The following stimulation conditions were used throughout the studies: cells were either prestimulated with zVAD (10 μM), Nec-1 (50 μM), TNFR2-Fc (30 μg/ml), TWEAK (0.5 μg/ml), or IAP antagonist (100 nM, unless otherwise specified) alone or in the respective combinations for 1 hr followed by stimulation with poly(I:C) (2 μg/ml for crystal violet assays, Annexin V externalization, HMGB-1 release, and immunofluorescence microscopy; or 20 μg/ml for IP).

Crystal violet staining of attached, living cells was performed 18–24 hr after stimulation exactly as described (Kavuri et al., 2011). For statistical analysis, the standard error of mean (SEM) was determined for three to five independent experiments of each cell line and stimulatory condition (represented by error bars in the figures). For the detection of phosphatidylserine externalization, cells were stimulated and analyzed exactly as previously described (Geserick et al., 2009). For analysis of HMGB-1 release, cells were stimulated for 18 hr. Cell-free supernatants and cellular lysates were analyzed for HMGB-1 protein expression by western blotting.

### Coimmunoprecipitation of Caspase-8- and cFLIP-Bound Complexes

For the precipitation of caspase-8-bound proteins and cFLIP-bound proteins, 1 × 10^7^ cells were used. Cells were prestimulated with either zVAD (10 μM) or IAP antagonist (100 nM) alone or the respective combinations thereof for 1 hr followed by stimulation with 20 μg/ml of poly(I:C) for 3 hr or HF-TNF for 2 hr. Details of the procedure are exactly as described (Geserick et al., 2009). For coimmunoprecipitation of Flag-tagged complexes, 40 μl of anti-Flag M2 agarose beads (Sigma, Germany) were added to lysates. Samples were incubated for 14–18 hr on an end-over-end shaker at 4°C. Precipitates were washed five times with lysis buffer before protein complexes were eluted from dried beads by addition of standard reducing sample buffer and boiling at 95°C. Equal amounts of precipitates were analyzed by western blotting (Schmidt et al., 2010; see also the Supplemental Experimental Procedures).

### Chromatographic Gel Filtration Assay

Cellular lysates were separated on a Superose 6 HR 10/30 size-exclusion column and an AKTA Purifier protein purification system (GE Healthcare) essentially as described previously (Cain et al., 2000). Lysates (3–4 mg of total protein) were separated at a flow rate of 0.4 ml/min, and 0.5 ml fractions were collected at 4°C in 5% (w/v) sucrose, 20 mM HEPES, 120 mM NaCl, 2 mM EDTA, 2 mM KCl, and 1% Triton X-100 (pH 7.5). The column was calibrated with the indicated protein standards (GE Healthcare, Figure 5F). For each sample, three separate column separations were performed and repeat fractions collected in the same tubes. Aliquots (100 μl) from each fraction were retained for western blotting. For IP, fractions were then pooled (1–5, 6–10, 11–16, 17–22, and 23–28), and IPs were performed as described above followed by western blot analysis.

## Supplemental Information

Document S1. Four Figures, Supplemental Experimental Procedures, and Supplemental References
