# Supplementary material for: cIAPs Block Ripoptosome Formation, a RIP1/Caspase-8 Containing Intracellular Cell Death Complex Differentially Regulated by cFLIP Isoforms
Source: Mol Cell. 2011 Aug 5;43(3-6):449–63. doi: 10.1016/j.molcel.2011.06.011 (PMC3163271; doi:10.1016/j.molcel.2011.06.011)
Supplement: Document S1. Four Figures, Supplemental Experimental Procedures, and Supplemental References [file mmc1.pdf]

Molecular Cell, Volume *43*

Supplemental Information

cIAPs Block Ripoptosome Formation, a RIP1/Caspase-8 Containing Intracellular Cell Death Complex Differentially Regulated by cFLIP Isoforms

Maria Feoktistova, Peter Geserick, Beate Kellert, Diana Panayotova Dimitrova, Claudia Langlais, Mike Hupe, Kelvin Cain, Marion MacFarlane, Georg Häcker, and Martin Leverkus

Inventory of Supplemental information

Figure S1, related to Fig. 1

IAP inhibition or TWEAK sensitize to TLR3-induced cell death in a death receptor-independent manner

Figure S2, related to Fig. 2

TLR3 and TRIF are critical for TLR3-induced cell death that proceeds in a RIP1-dependent manner

Figure S3, related to Fig. 4

Qualitative responses to TLR3 ligation and the role of cFLIP isoforms for Ripoptosome formation in transformed epithelial cell lines and the impact of RIP3

Figure S4, related to Fig. 5

Kinetic responses to IAP antagonist in $cFLIP_S$ expressing HaCaT and analysis of TNF-induced complex I and II formation in the absence of cIAPs in HaCaT

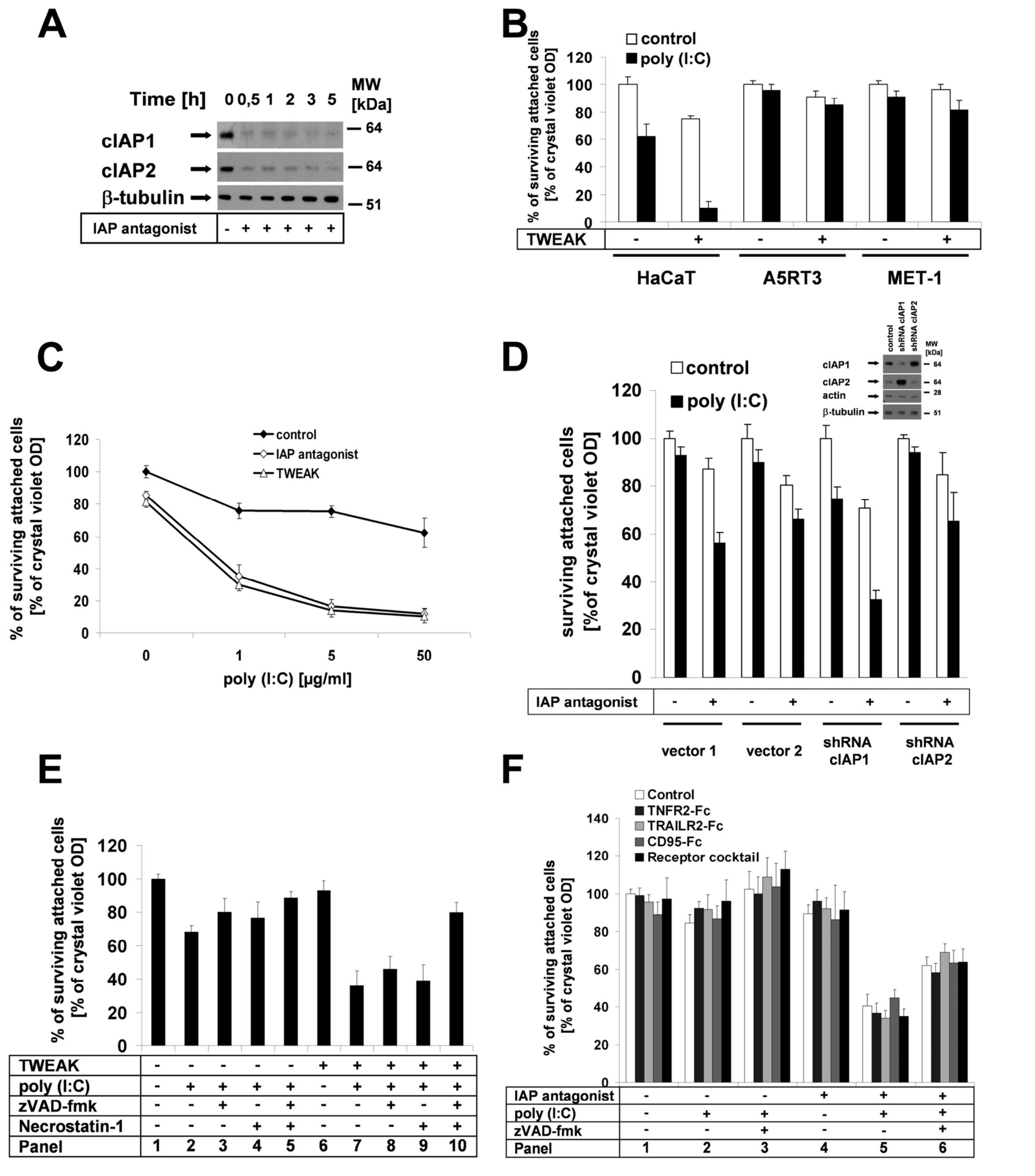

Figure S1. TWEAK- or siRNA-mediated knockdown of cIAP1, but not cIAP2 sensitizes for TLR3-induced cell death independent of death receptor autocrine loops, Related to Figure 1.

A-F. Cells were stimulated with TWEAK and IAP antagonist as described in Experimental procedures. **A.** IAP antagonist rapidly reduces cellular levels of cIAP 1 and 2. The cells were pre-treated with IAP antagonist for indicated time periods, followed by Western blot analysis **B-F:** Cell viability was analyzed by crystal violet assay; mean of 3 independent experiments (±SEM) are shown. **B.** TWEAK sensitizes HaCaT but not A5RT3 or MET-1 cells to TLR3-induced cell death. poly (I:C) concentration was [50µg/ml]. **C.** Dose-dependent response of HaCaT cells to poly (I:C) in presence of IAP antagonist or TWEAK. **D.** Knockdown of cIAP1, but not cIAP2 sensitizes for TLR3-mediated cell death. HaCaT cells were transduced with cIAP1 or cIAP2 shRNA or vector controls. cIAP1 and cIAP2 expression was investigated by Western blot analysis (inset). **E.** TWEAK sensitizes HaCaT cells to TLR3 mediated caspase-

and RIP1 kinase-dependent cell death. **F.** TLR3 induced cell death in the absence of IAP function is independent of death receptor autocrine loops. HaCaT were pre-treated for 1h with either TNFR2-Fc (30 µg/ml), TRAILR2-Fc (30µg/ml), CD95-Fc (50% of a cellular supernatant) or a combination thereof. Cells were then stimulated with zVAD-fmk and IAP antagonist, followed by treatment with poly (I:C).

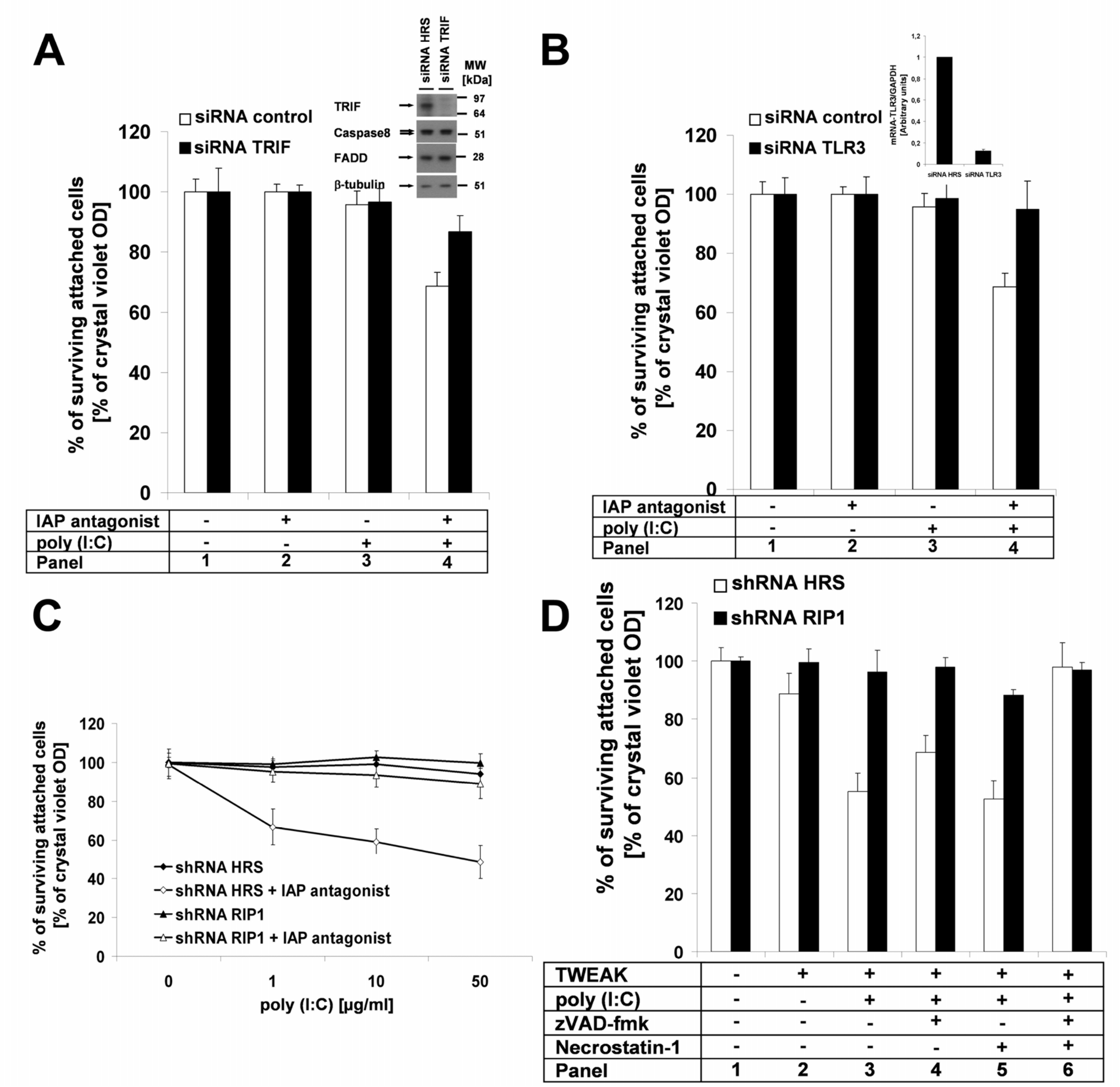

Figure S2. Poly (I:C)-induced cell death requires TLR3, TRIF, and RIP1, Related to Figure 2

A-D: Cells were stimulated as described in Experimental procedures. Cell viability was analyzed by crystal violet assay; average values of 3 independent experiments (±SEM) are shown. **A.** TLR3 induced cell death in the absence of cIAPs is TRIF-dependent. HaCaT cells were transfected with control or TRIF siRNA. TRIF knockdown was analyzed by Western blotting (inset). **B.** TLR3-induced cell death in the absence of cIAPs is TLR3-dependent. HaCaT cells were transfected with TLR3 siRNA or control siRNA. TLR3 knockdown was confirmed by qPCR average values of 2 independent experiments (±SEM) are shown (inset). **C and D:** HaCaT were transduced with control (HRS) or RIP1 specific shRNA. **C.** Dose-dependent response of cells to poly (I:C) in the presence or absence of IAP antagonist was investigated. **D.** TWEAK-induced sensitization to TLR3-induced cell death critically requires RIP1.

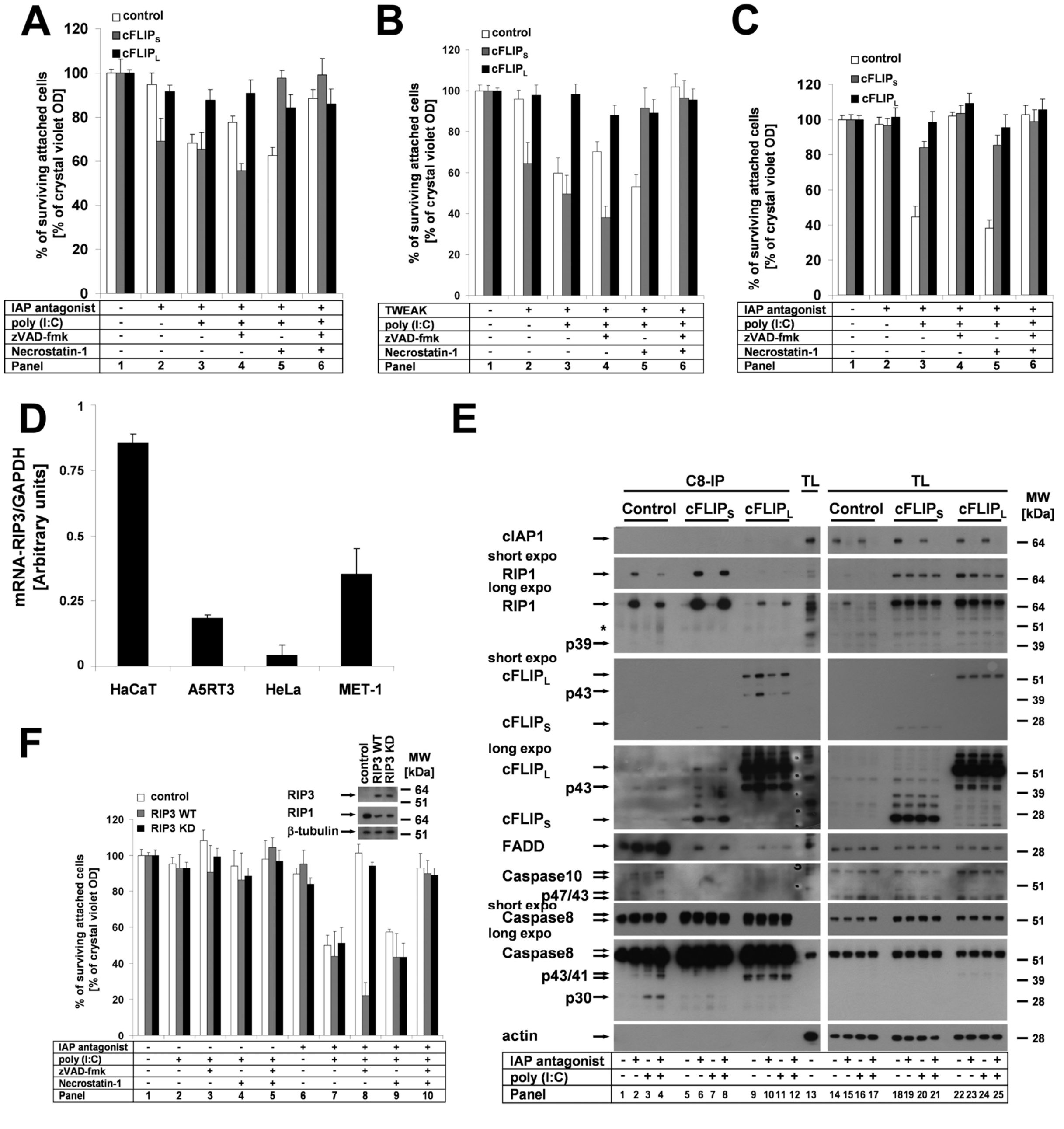

Figure S3. cFLIP$_L$, but not cFLIP$_S$ protects from Ripoptosome-induced RIP3-dependent necroptosis, Related to Figure 4.

A-C and E. Experiments were performed in HaCaT (A and B) and HeLa (C, E, and F) cells transduced with cFLIP$_L$ or cFLIP$_S$ or vector alone. **A-C, F:** Cells were treated as described in Experimental procedures. Cellular viability was analyzed by crystal violet assay; average values of 3 independent experiments (±SEM) are shown. **A.** cFLIP$_L$, but not cFLIP$_S$ protects HaCaT from TLR3-induced cell death in the presence of IAP antagonist. **B.** cFLIP$_L$, but not cFLIP$_S$ protects HaCaT from TLR3-induced cell death in the presence of TWEAK. **C.** cFLIP$_L$, and cFLIP$_S$ protect HeLa from TLR3-induced cell death in the presence of IAP antagonist. **D.** HeLa cells have the least amounts of RIP3 from all studied cell lines. RIP3 mRNA levels were analyzed by qPCR in HaCaT, A5RT3, MET-1, and HeLa cells, average values of 2 independent experiments (±SEM) are shown. **E.** Ripoptosome formation in HeLa cells is blocked by cFLIP$_L$, but promoted by cFLIP$_S$. Cells were pre-treated with zVAD-fmk (10 μM) and IAP antagonist (200 nM) for 1h and stimulated with poly (I:C) for 2h and assayed for Ripoptosome formation by caspase 8 IP. **F.** The impact of RIP3 for qualitative cell death responses in Hela. Re-expression of RIP3 wildtype (RIP3 WT) but not of the RIP3 kinase deficient mutant (RIP3 KD) in Hela cells promotes a switch from caspase-dependent to

caspase-independent cell death. RIP3 WT and RIP3 KD expression was analyzed by Western blotting (inset).

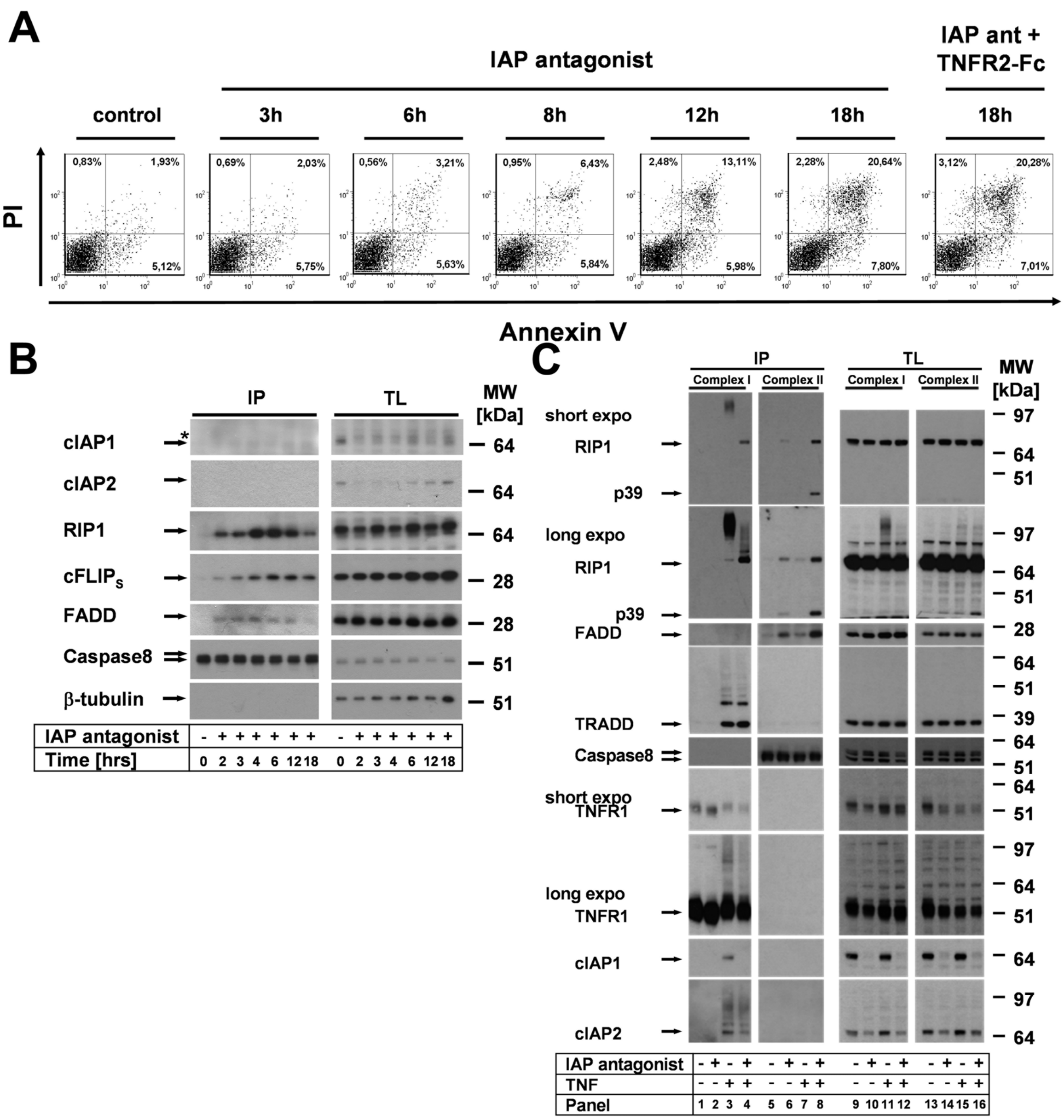

Figure S4. cIAPs block time-dependent formation of the Ripoptosome in HaCaT-cFLIP$_S$ and modulate TNF complex I and II formation in HaCaT, Related to Figure 5.

A. Time kinetic of spontaneous cell death in HaCaT-cFLIP$_S$ in the absence of cIAPs. Cells were treated with IAP antagonist and TNFR2-Fc for the indicated times, followed by AnnexinV/PI double staining and FACS analysis. **B.** Time kinetics of spontaneous Ripoptosome formation in HaCaT-cFLIP$_S$ in the absence of cIAPs. HaCaT-cFLIP$_S$ were treated with zVAD-fmk (10 µM) and IAP antagonist (200 nM) for the indicated times and assayed for Ripoptosome formation by caspase 8 IP. Asterisks indicate non-specific binding of Abs. **C.** TNF-induced receptor signalling complexes in HaCaT keratinocytes. HaCaT were stimulated with 2,5 µg/ml TNF for 10 min (complex I) or 2 hrs (complex II). TNF Complex I was precipitated by ligand affinity precipitation using HF-TNF as described (Diessenbacher et al., 2008). Caspase 8 IP was performed as described in Experimental procedures. Precipitation of TNFR1 following lysis (-) with HF-TNF served as internal specificity control when comparing to ligand affinity precipitates (+). Equal amounts of total cellular lysates (lysates) or ligand affinity-precipitates (IP) were analyzed by Western blotting for the indicated molecules.

Supplemental Experimental Procedures

Materials

The following antibodies (Abs) were used for Western blot analysis: Abs to caspase 8 (C-15; kindly provided by P.H. Krammer, C-20; Santa Cruz, Delaware Avenue, California), cFLIP (NF-6; Alexis, San Diego, California), rabbit RIP3 Abs (generously provided by F. K. Chan), FADD, XIAP (#62120), Caspase 10 (MBL, Nagoya, Japan), TRADD and RIP1 (Transduction Laboratories, San Diego, California), TNFR1 (Santa Cruz, Delaware, California), rat Abs to cIAP1 (Silke et al., 2005) and cIAP2 (Vince et al., 2009). TRIF Abs were purchased from Cell Signaling Technology (#4596). Polyclonal Abs to HMGB1 (ab18256) were purchased from Abcam, β–actin and β-tubulin (clone 2.1) Abs were from Sigma (Saint Louis, Missouri, USA). TNFR2-Fc was obtained from Wyeth Pharmaceuticals. For the production of recombinant Fc-TWEAK we used a construct kindly provided by P. Schneider, Epalinges, Switzerland as published (Geserick et al., 2009). Horseradish peroxidase (HRP)-conjugated goat anti-rabbit, goat anti rat IgG, goat anti-mouse IgG Abs and HRP-conjugated goat anti-mouse IgG1, IgG2a, IgG2b, and IgG1κ were obtained from Southern Biotechnology Associates (Birmingham, AL). Poly (I:C), Necrostatin-1 and MG-132 were purchased from Sigma (Saint Louise, Missouri, USA). The IAP antagonist (Compound A) was kindly provided by TetraLogics Pharmaceuticals (Philadelphia, USA). Pan-caspase inhibitor Z-Val-Ala-DL-Asp-fluoromethylketone (zVAD-fmk) was purchased from Bachem GmbH (Germany). HF-TNF was prepared and used as described (Diessenbacher et al., 2008).

Cell culture

The spontaneously transformed keratinocyte line HaCaT and the derived metastatic clone A5-RT3 were provided by Dr. Petra Boukamp (DKFZ, Heidelberg). Both cell lines do not express XIAP (Diessenbacher et al., 2008). MET-1 cells that express high levels of XIAP protein were provided by I. Leigh, Skin Tumor Laboratory, Cancer Research UK, London, UK). Cell lines were exactly cultured as described (Geserick et al., 2009). HeLa cells were provided by Dr. Michael Boutros (DKFZ, Heidelberg) and were cultured in RPMI medium containing 10 % FCS (fetal calf serum). Primary human keratinocytes (PK) from different donors were cultured in PK medium from CELLnTEC Advanced Cell Systems (Bern, Switzerland). All experiments were performed between passages 3-8 after primary cell culture.

Real-Time quantitative PCR (RT qPCR)

RNA isolation was performed with RNeasy Kit (Qiagen, Hilden, Germany). cDNA was synthesized in 20µl using a mixture of random nonamers and oligo dT primers in a ratio of 10:1 and SuperScript II Reverse Transcriptase (Invitrogen, California, USA). Primers were designed using Primer3 software (available at ttp://frodo.wi.mit.edu/cgibin/primer3/primer3_www.cgi). RT qPCR analyses for the genes encoding TLR3, RIP3 and glyceraldehyde-3-phosphate dehydrogenase (GAPDH) was performed in a final volume of 20µl using KAPA SYBR Fast qPCR (Peqlab, Erlangen, Germany) in the Mx3005P (Stratagene, LaJolla, CA, USA) real time termal cycler. The following primers were used:

TLR3
forward 5'- TCCAACAGAATCATGAGACA -3';
reverse 5'- ATGGGTAAGGTTCAACACTG -3';
RIP3
forward 5'- CAAGATCGTAAACTCGAAGG -3';

reverse 5'- CCGTTCTCCATGAATTTAGT;
GAPDH
forward 5'- CCTGGTATGACAACGAATTT -3';
reverse 5'- GTGAGGGTCTCTCTCTTCCT -3'.
Equal cycling conditions were used to amplify the genes of interest and reference gene products. KAPA SYBR Fast DNA Polymerase was launched by an initial 15 min at 95°C followed by 42 cycles of one step (denaturation) at 94°C for 15 sec, one step (annealing) at 55°C for 30sec, and one step (extension) at 72°C for 30sec. Melting curve analysis was used to confirm the specific amplification of a single product of the expected size for each gene. Serial dilutions of cDNA (1, 1/5, and 1/25) were amplified for the construction of a standard curve (plotted as a logarithmic function of the cDNA dilution factor) and used for the estimation of the RT PCR efficiency using Mx3005P software. The relative quantification for TLR3 or RIP3 was calculated after dividing the standard curve value of the respective genes by that of the reference gene (GAPDH) for each individual sample. The effects on TLR3 or RIP3 expression were calculated by analyzing mean values obtained from two independent experiments. In the two independent experiments, RNA was reverse transcribed three times each and the pooled cDNAs from three independent reverse transcriptions were assayed for RT qPCR in triplicates. The mean values obtained by the above explained procedure were compared for all different experimental conditions. The error bars in the figures represent SEM.

Immunofluorescence microscopy

For detection of nuclear morphology and integrity of the cell membrane, $5x10^4$ cells of the respective cells were seeded per well in a 12-well plate. Following 24 hrs of incubation for adherence, cells were stimulated as previously described for 24 hrs. Subsequently, cells were incubated with Hoechst 33342 (5µg/ml; Polysciences Europe, Eppelheim, Germany) and SYTOX® Green (5pM; Invitrogen™, Molecular Probes™, Eugene, Oregon, USA) for 15 min at 37°C immediately followed by phase contrast or fluorescence microscopy using a Zeiss HBO 50 microscope (Jena, Germany). Digital images were processed in an identical manner using the advanced SPOTSOFTWARE, Version 4.6 (Diagnostic Instruments Inc, MI, USA).

Stable expression and transient transfection of siRNA

We used stable retroviral expression of shRNA for knockdown of cIAP1 (in pLKO.1 vector, Open Biosystems), cIAP2, cFLIP, or RIP1 in pRS-based constructs as described (Diessenbacher et al., 2008; Geserick et al., 2008). A shRNA of a hyper random sequence (HRS) not matched by any gene in the NCBI database was used as a control (Geserick et al., 2009). To generate lentiviral supernatants, 293T cells were transfected with 3 µg pMD2.G, 5 µg pMDlg/pRRE, and 2,5 µg pRSV-Rev of the packaging vectors together with the constructs described above. Cells were infected with virus-containing supernatants, were selected with puromycin (1 µg/ml; Sigma, Taufkirchen, Germany) for 3 days and expression of GFP was analyzed (always >90%, data not shown) by FACS. To confirm knockdown of the respective molecules at the protein level Western blot analysis was performed. Aliquots of cells were used for the experiments between passages 2-6 for all subsequent studies. For transient knockdown experiments the following siRNA duplexes was used: FlexiTube siRNA for Caspase 8 (Hs_CASP8_11), TNFR1 (Hs_TNFRSF1A_5) and RIP3 (Hs_RIPK3_1) and the respective control siRNA (AllStars neg. control siRNA 1027281) and were from QIAGEN (Hilden, Germany). TRIF (TICAM1HSS152364) and TLR3 (TLR3HSS110817) siRNA duplexes were from Invitrogen (Karlsruhe, Germany). For transient transfection $2x10^5$ cells were seeded per well and incubated overnight at 37°C. Prior to transfection cells were incubated with Opti-MEM (Gibco 11058-021) medium for 20 min followed by transfection

according to the manufacturer´s recommendations using Lipofectamine 2000 (Invitrogen, #11668019) and the respective siRNA species.

Generation of cFLIP and RIP3 overexpressing cell lines

For retroviral cFLIP and RIP3 expression in HaCaT and HeLa cells, transfection and transduction protocols were used exactly as previously reported (Geserick et al., 2008). FACS analysis of GFP expression and Western blot analysis was performed on polyclonal cells after a 10 – 14 day selection period to confirm ectopic protein expression. For overexpression of RIP3 WT and RIP3 KD (D160N) the corresponding cDNAs were subcloned from pEGFP N1 vectors into pCFG5-IEGZ retroviral vector by standard cloning procedures and verified by sequencing. For overexpression of Flag-tagged cFLIP isoforms the cDNAs of $cFLIP_S$ or $cFLIP_L$ were subcloned into the TWEEN vector that contains the sequence for the Flag-Epitope at the N-terminal region of the protein (Haas et al., 2009). Lentiviral supernatants were produced exactly as described (Diessenbacher et al., 2008). Virus was added to HaCaT cells together with 1 µg/ml polybrene (Sigma H9268). GFP expressing cells were sorted by FACSAria cell sorter (Becton Dickinson & Co, San Jose, CA) and expression of Flag-tagged cFLIP proteins was confirmed by Western blotting.

Western blot analysis

Cell lysates were prepared as described (Diessenbacher et al., 2008) and 5 µg of total cellular proteins were separated by SDS-PAGE on 4-12% gradient gels (Invitrogen, Karlsruhe, Germany) followed by transfer to nitrocellulose or PVDF membranes. Blocking of membranes and incubation with primary and appropriate secondary Abs (Supplemental Experimental Procedures) were essentially performed as described previously (Geserick et al., 2009). Bands were visualized with ECL detection kits (Amersham, Freiburg, Germany).

Supplemental References

Diessenbacher, P., Hupe, M., Sprick, M.R., Kerstan, A., Geserick, P., Haas, T.L., Wachter, T., Neumann, M., Walczak, H., Silke, J., and Leverkus, M. (2008). NF-kappaB inhibition reveals differential mechanisms of TNF versus TRAIL-induced apoptosis upstream or at the level of caspase-8 activation independent of cIAP2. J Invest Dermatol 128, 1134-1147.

Geserick, P., Drewniok, C., Hupe, M., Haas, T.L., Diessenbacher, P., Sprick, M.R., Schon, M.P., Henkler, F., Gollnick, H., Walczak, H., and Leverkus, M. (2008). Suppression of cFLIP is sufficient to sensitize human melanoma cells to TRAIL- and CD95L-mediated apoptosis. Oncogene 27, 3211-3220.

Geserick, P., Hupe, M., Moulin, M., Wong, W.W., Feoktistova, M., Kellert, B., Gollnick, H., Silke, J., and Leverkus, M. (2009). Cellular IAPs inhibit a cryptic CD95-induced cell death by limiting RIP1 kinase recruitment. J. Cell Biol. 187, 1037-1054.

Haas, T.L., Emmerich, C.H., Gerlach, B., Schmukle, A.C., Cordier, S.M., Rieser, E., Feltham, R., Vince, J., Warnken, U., Wenger, T., Koschny, R., Komander, D., Silke, J., and Walczak, H. (2009). Recruitment of the linear ubiquitin chain assembly complex stabilizes the TNF-R1 signaling complex and is required for TNF-mediated gene induction. Mol. Cell 36, 831-844.

Silke, J., Kratina, T., Chu, D., Ekert, P.G., Day, C.L., Pakusch, M., Huang, D.C., and Vaux, D.L. (2005). Determination of cell survival by RING-mediated regulation of inhibitor of apoptosis (IAP) protein abundance. Proc. Natl. Acad. Sci. U. S. A 102, 16182-16187.

Vince, J.E., Pantaki, D., Feltham, R., Mace, P.D., Cordier, S.M., Schmukle, A.C., Davidson, A.J., Callus, B.A., Wong, W.W., Gentle, I.E., Carter, H., Lee, E.F., Walczak, H., Day, C.L., Vaux, D.L., and Silke, J. (2009). TRAF2 must bind to cellular inhibitors of apoptosis for tumor necrosis factor (tnf) to efficiently activate nf-{kappa}b and to prevent tnf-induced apoptosis. J. Biol. Chem. 284, 35906-35915.
